# Refractory septic shock: our pragmatic approach

**DOI:** 10.1186/s13054-018-2144-4

**Published:** 2018-09-19

**Authors:** Prashanth Nandhabalan, Nicholas Ioannou, Christopher Meadows, Duncan Wyncoll

**Affiliations:** Department of Critical Care, St Thomas’ Hospital, Westminster Bridge Rd., Lambeth, London, SE1 7EH UK

**Keywords:** Refractory septic shock, Multi-organ failure, High-dose vasopressors, Septic cardiomyopathy, Toxin-producing bacteria

## Abstract

Despite timely intervention, there exists a small subgroup of patients with septic shock who develop progressive multi-organ failure. Seemingly refractory to conventional therapy, they exhibit a very high mortality. Such patients are often poorly represented in large clinical trials. Consequently, good evidence for effective treatment strategies is lacking. In this article, we describe a pragmatic, multi-faceted approach to managing patients with refractory septic shock based on our experience of toxin-mediated sepsis in a specialist referral centre. Many components of this strategy are inexpensive and widely accessible, and so may offer an opportunity to improve outcomes in these critically ill patients.

## Introduction

The Surviving Sepsis Guidelines provide a suitable framework to guide therapy for the majority of patients with septic shock [1]. Appropriate and timely antimicrobial therapy, source control if indicated, fluid therapy, and targeted vasopressors remain the backbone of treatment. However, a small proportion of patients fail to respond to these measures and deteriorate precipitously into refractory shock and progressive multi-organ failure. This subgroup of patients is often poorly represented in large randomised controlled trials investigating the efficacy of interventions in septic shock. As a result, there is little conclusive evidence to guide management in this particular population.

Refractory septic shock is variably defined as the presence of hypotension, with end-organ dysfunction, requiring high-dose vasopressor support often greater than 0.5 μg/kg/min norepinephrine or equivalent [2]. Regardless of the precise definition, there is an associated mortality of up to 60%. Furthermore, patients with vasopressor requirements greater than 1 μg/kg/min norepinephrine or equivalent who continue to deteriorate clinically have a reported mortality as high as 80–90% [3, 4]. Microcirculatory failure and associated ischaemic consequences are frequently observed and alternative therapeutic strategies are desperately needed to improve outcomes in this small subgroup of critically ill patients.

In this viewpoint article we describe a pragmatic, multi-faceted approach to managing patients with refractory septic shock. The list of interventions described below is drawn from our clinical experience managing patients with confirmed, or suspected, toxin-producing bacteria in a specialist Severe Respiratory Failure centre in the UK. It is recognised that some of these interventions lack a robust evidence base. Our intention is not to rehearse the current evidence for each component of therapy, but merely to describe our institutional approach with brief reference to selected relevant literature.

### Albumin

Early fluid requirements in these patients often significantly exceeds the standard recommended initial regimen of 30 ml/kg. Our practice is to use balanced crystalloids for initial volume replacement, guided by dynamic cardiac output monitoring and echocardiography, followed by 20% human albumin solution if ongoing fluid resuscitation is required. During the early phase of severe shock we target a serum albumin level of > 30 g/l. Albumin maintains plasma oncotic pressure and acts as an antioxidant and as a buffer for acid-base equilibrium. Although conclusive proof for resuscitation with albumin is lacking, a subgroup analysis of 1121 patients with septic shock in the ALBIOS trial demonstrated a reduced mortality [5]. Other studies have also suggested a beneficial effect. However, debate continues over the role of albumin in septic shock with concerns mainly related to cost-effectiveness [6]. Our approach is informed by physiological rationale, a suggestion of benefit in clinical studies, and limited evidence for harm associated with albumin administration.

### Hydrocortisone

The use of corticosteroids in septic shock has been frequently studied. It has been argued that steroid treatment reduces the duration of shock and length of intensive care unit (ICU) stay [7]. Large randomised controlled trials have failed to identify a clear survival benefit [8]. However, the beneficial effects may only be seen in those patients with the highest illness severity scores [9]. Our practice is to administer a hydrocortisone infusion (8 mg/h following a 50-mg bolus) to all patients with refractory septic shock on the basis that these patients are most likely to benefit and there is little evidence of harm. This is supported by results from the recently published APPROCHS study [10] where a survival benefit was seen in a population of septic shock patients with high mortality (43.0% vs 49.1% in controls). This compares to no difference in outcome in the ADRENAL study where the observed mortality was much lower (27.9% vs 28.8% in controls) [11].

### Femoral arterial access

Radial arterial pressure waveforms often underestimate blood pressure in the context of severe hypovolaemia and peripheral vasoconstriction. This can lead to the administration of significantly higher doses of vasopressor to achieve the ‘target mean arterial blood pressure (MAP)’. In early septic shock, the difference between radial and femoral invasive MAP measurements is reported to be around +5 mmHg; however, this discrepancy is increased in advanced shock [12]. We routinely use femoral arterial access for invasive blood pressure monitoring in this population. The subsequent increase in measured MAP frequently allows a significant reduction in vasopressor dosing in a considerable proportion of patients [13].

### Lower the MAP target

Although retrospective analyses of haemodynamic variables are available [14], the traditional MAP target of 65 mmHg has not been subjected to scrutiny by many randomised controlled trials. In a recently published pooled analysis, lower blood pressure targets were not associated with adverse outcomes even in patients with pre-existing hypertension [15]. Individually selected goals are likely to be more appropriate than rigid prescriptive targets. Arguably, preservation of renal function is less vital as patients with refractory septic shock are often already receiving renal replacement therapy. Furthermore, splanchnic perfusion has been shown to be adequate with a MAP target above 50 mmHg if hypovolaemia is avoided in selected patient groups [16]. Young, previously well patients are particularly tolerant of lower systemic blood pressure. We therefore reduce the MAP target in patients with refractory septic shock to 50–55 mmHg. Our experience is that, in selected patients without intracranial pathology, this lower MAP target allows a worthwhile reduction in vasopressor requirements leading to improved tissue perfusion and an associated reduction of hyperlactataemia. Norepinephrine remains our vasopressor of choice and we avoid the use of vasopressin which, in our experience, appears to be associated with an increased risk of peripheral and mesenteric ischaemia in patients with refractory septic shock. Early enteral nutrition is also avoided in these patients with refractory septic shock on high-dose vasopressors; we prefer the use of parenteral nutrition until the shock state has resolved.

### Minimise sedation

Sedative medications exacerbate hypotension through myocardial depression and systemic vasodilation. Microcirculatory flow may also be impaired. Current guidelines suggest minimising sedation in mechanically ventilated patients with sepsis [1]. However, our experience is that this approach is not always adhered to. Patients with refractory septic shock often have a reduced level of consciousness as a result of septic encephalopathy, and consequently sedation requirements may be even lower than the general ICU sepsis population. Furthermore, altered hepatic metabolism and reduced renal clearance may lead to accumulation of sedative agents in shocked patients [17]. Sedative strategies and agents are numerous. Perfusion may be improved using low-dose midazolam instead of propofol [18]. However, delirium, accumulation, and duration of action can limit the usefulness of long-term benzodiazepine infusion. We minimise sedation in patients with refractory septic shock. Where sedation is required, our first-line strategy is to use a predominantly opiate-based regimen in conjunction with low-dose propofol titrated to a specified target sedation score.

### Replacement of thiamine and vitamin C

Vitamin C (ascorbic acid) is an essential water-soluble substance that cannot be synthesised by the body. It has powerful antioxidant properties and functions as an important enzyme co-factor in the biosynthesis of endogenous catecholamines and vasopressin [19]. It also enhances host defence mechanisms by improving macrophage and T-cell immunity. Levels of vitamin C remain extremely low in critically ill patients despite regular supplementation. This is exacerbated in patients with septic shock where vitamin C deficiency is common despite achieving targeted intake via enteral or parenteral nutrition [20]. In a phase I study, high-dose intravenous vitamin C reduced organ failures and pro-inflammatory plasma biomarkers in severe sepsis with no reported adverse effects [21]. Others have reported a significant reduction in vasopressor requirements with intravenous replacement of vitamin C [22]. Further trials are ongoing, but intravenous replacement of vitamin C in septic shock is based on scientific rationale and appears to be a safe and useful intervention [23].

Vitamin B1 (thiamine) is a water-soluble vitamin with an essential role in carbohydrate metabolism and energy production. Absolute or relative thiamine deficiency is common in patients with septic shock [24]. Such a deficiency may present as an unexplained lactic acidosis but remains undetected since routine red cell transketolase measurements are rarely available and often very costly. Intravenous thiamine replacement has been shown to reduce lactate levels and mortality in patients with proven thiamine deficiency [25]. Furthermore, intravenous thiamine replacement may also be associated with a reduced need for renal replacement therapy and improved renal function in patients with septic shock [26].

Our practice is to give combined vitamin C (4.5 g/day) and thiamine (2.25 g/day) using three pairs of intravenous Pabrinex™ three times per day until shock has resolved. This dosing regimen has been used in our institution, hospital-wide, for several years to prevent Wernicke’s encephalopathy in alcoholics. Combination therapy may be more effective with the suggestion of a synergistic effect between the two agents [27]. A recent retrospective cohort study demonstrated a dramatic reduction in organ failures, duration of vasopressor support, and mortality using combination treatment with intravenous hydrocortisone, vitamin C, and thiamine [28]. The presence of thiamine may mitigate concerns over renal oxalate crystal precipitation secondary to high-dose vitamin C and, whilst more robust evidence is awaited, there appears to be little harm with this approach.

### Adjunctive antimicrobial therapy

In addition to broad spectrum antibiotics, we routinely administer clindamycin to patients with refractory septic shock until initial microbiological analyses have excluded toxin-producing pathogens or until stabilisation of organ dysfunction is achieved. Clindamycin inhibits bacterial protein synthesis and prevents generation of super-antigens. It is an inexpensive and accessible intervention with a proven efficacy in toxic shock syndrome [29]. Although recommended by several guidelines, clindamycin is often considered late into a patient’s presentation despite maximal benefit being associated with early administration.

### Intravenous immunoglobulin (IVIG)

Treatment with IVIG in patients with septic shock has been proposed for several decades. There is extensive biological plausibility as to the beneficial immunological effects of IVIG in patients with toxin-mediated septic shock [30]. However, the literature remains conflicting, with several meta-analyses failing to demonstrate improved outcomes. Although current guidelines recommend against the routine use of IVIG in septic shock, it is acknowledged that further trials are needed. Early administration is likely to offer the optimal prospect of benefit. We empirically initiate treatment with IVIG to progressively deteriorating patients with refractory septic shock secondary to suspected toxin-producing organisms such as group A streptococcus (1 g/kg on day 1, then 0.5 g/kg on days 2 and 3) or Panton-Valentine leukocidin (PVL) *Staphylococcus aureus* (2 g/kg on day 1, repeated on day 3 if no improvement).

### Levosimendan

Septic cardiomyopathy resulting in a low cardiac output state is relatively common in patients with refractory septic shock. Central venous saturations (ScvO_2_) may be difficult to interpret in this context due to significant impairment of oxygen utilisation. Screening echocardiography identifies those patients with moderate to severely impaired myocardial function and may exclude primary cardiogenic causes. Dobutamine has traditionally been used in this context, but exacerbation of existing tachycardia and increased myocardial oxygen consumption limit its usefulness. Alternatively, improved cardiac function can be achieved using levosimendan in conjunction with the maintenance of ionised calcium levels greater than 1.2 mmol/l. Although the LeoPARDS trial found no benefit with levosimendan in patients with sepsis [31], it is difficult to extrapolate these findings to a subgroup with refractory shock. Only 10% of the patients studied demonstrated evidence of a low cardiac output state and mortality was much lower than would be expected in this subgroup. Our practice is to administer levosimendan to patients with echocardiographic features of moderate to severely impaired left ventricular systolic function and impaired end-organ perfusion. Concerns over the potential need for increased vasopressor requirements can be mitigated by many of the points previously described in this article.

### Epoprostenol and heparin

Intravenous prostacyclin has beneficial effects on microcirculatory flow. It has been shown to increase oxygen delivery in critically ill patients [32] and successfully reverse symmetrical peripheral limb ischaemia secondary to high-dose vasopressors in septic shock [33]. Its wider use is frequently limited by concerns over exacerbating hypotension; other vasodilators such as nitrates are used by other centres, but in our experience do not appear to be as effective. In patients with refractory septic shock with peripheral mottling we commence a low-dose epoprostenol infusion (0.5–5 ng/kg/min) to improve microcirculatory flow and prevent the occurrence of peripheral thrombotic events. Our experience is that peripheral ischaemic complications are reduced and haemodynamic compromise is rarely encountered if the prostacyclin infusion is titrated up very slowly*.* In the setting of disseminated intravascular coagulation and suspicion of end-organ microthrombosis, and in the absence of absolute contra-indications, we also initiate low-dose intravenous heparin infusion (fixed rate 250–500 IU/h).

### Renal replacement therapy

Although the IVOIRE study did not identify a survival benefit with high-volume haemofiltration compared with standard dosing [34], in refractory septic shock our practice is to initiate early haemodiafiltration with doses of 40–60 ml/kg/h. This facilitates rapid temperature control and correction of metabolic acidosis which, in our experience, contributes to a reduction in vasopressor requirements and improved cardiac output. Whilst there are concerns about removal of antibiotics, water-soluble vitamins, and trace elements, a recent review concluded that high-volume haemofiltration is not associated with adverse effects [35]. Appropriate compensatory antibiotic dosing and vitamin/trace element supplementation must be taken into account. Correction of metabolic acidosis may be achieved with sodium bicarbonate [36] but this risks further fluid administration and sodium overload, both of which can be avoided with renal replacement therapy.

### Extracorporeal support

Finally, in highly selected patients with refractory septic shock (often in the context of severe respiratory failure), extracorporeal technology providing respiratory and/or cardiac support achieves stability and buys time for the therapeutic interventions described above to have an impact. The benefits of extracorporeal support include improved global oxygen delivery, reduced intrathoracic pressures from reduced mechanical ventilatory requirements, improved carbon dioxide clearance and acid-base management, and improved myocardial performance. A recent publication has reported positive clinical outcomes using this approach [37].

## Conclusion

The management of refractory septic shock remains extremely challenging. We believe that where established conventional interventions fail to deliver improvements, a different approach using pragmatic strategies is necessary. Many of the interventions described here have proven biological plausibility but lack conclusive evidence. However, many remain unstudied in the context of refractory septic shock. Most of these strategies are relatively inexpensive, widely accessible, and are likely to be available in the majority of institutions. Our collective belief is that a bespoke approach can help to achieve haemodynamic stability and reverse progressive deterioration in this small subgroup of critically ill patients with a very high mortality.
